# Clinical ethics issues in HIV care in Canada: an institutional ethnographic study

**DOI:** 10.1186/s12910-017-0171-3

**Published:** 2017-02-06

**Authors:** Chris Kaposy, Nicole R. Greenspan, Zack Marshall, Jill Allison, Shelley Marshall, Cynthia Kitson

**Affiliations:** 1 Faculty of Medicine, Health Sciences Centre, Memorial University, St. John’s, NL A1B 3V6 Canada; 2 Present Address: St. Michael’s Hospital, 30 Bond St., Toronto, ON M5B 1W8 Canada; 3 Present Address: Renison University College, University of Waterloo, 240 Westmount Rd. N., Waterloo, ON N2L 3G4 Canada; 4 Winnipeg Regional Health Authority, Population and Public Health, 496 Hargrave St., Winnipeg, MB R3G 0X7 Canada; 5 Faculty of Health Sciences, School of Nursing, University of Ottawa, 451 Smyth Road, Ottawa, ON K1H 8M5 Canada

**Keywords:** Adherence, Bioethics, Clinical ethics, Confidentiality, HIV, Institutional ethnography, Nondisclosure, Social determinants

## Abstract

**Background:**

This is a study involving three HIV clinics in the Canadian provinces of Newfoundland and Labrador, and Manitoba. We sought to identify ethical issues involving health care providers and clinic clients in these settings, and to gain an understanding of how different ethical issues are managed by these groups.

**Methods:**

We used an institutional ethnographic method to investigate ethical issues in HIV clinics. Our researcher conducted in-depth semi-structured interviews, compiled participant observation notes, and studied health records in order to document ethical issues in the clinics, and to understand how health care providers and clinic clients manage and resolve these issues.

**Results:**

We found that health care providers and clinic clients have developed work processes for managing ethical issues of various types: conflicts between client-autonomy and public health priorities (“treatment as prevention”), difficulties associated with the criminalization of nondisclosure of HIV positive status, challenges with non-adherence to HIV treatment, the protection of confidentiality, barriers to treatment access, and negative social determinants of health and well-being.

**Conclusions:**

Some ethical issues resulted from structural disadvantages experienced by clinic clients. The most striking findings in our study were the negative social determinants of health and well-being experienced by some clinic clients – such as experiences of violence and trauma, poverty, racism, colonization, homelessness, and other factors affecting well-being such as problematic substance use. These negative determinants were at the root of other ethical issues, and are themselves of ethical concern.

## Background

AIDS emerged in the 1980’s as a novel illness and life-threatening disease. Because of the combination of the severity of disease, its transmissibility, and discrimination against the groups most affected by HIV, the emergence of AIDS gave rise to many challenging clinical ethics issues. Intense debates about the refusal of care played out in medical ethics journals and amongst the membership of professional groups such as doctors and nurses [1–5]. The protection of confidentiality became a contentious issue [6, 7]. Physicians and other caregivers wondered about their duty to warn third parties who might be at risk because of the activities of patients they knew were HIV-positive [8, 9]. Clinical ethics issues intersected with research ethics issues [7, 10] as well as with public health strategies for managing infectious disease outbreaks [11, 12].

The late 1990’s brought new treatments in the form of anti-retroviral therapy (ART), particularly the advent of protease inhibitors [13]. These treatments have changed the experience of living with HIV for many people, and have also altered the way HIV is perceived. ART has extended the lives of many who are living with HIV. Death rates declined and many people living with HIV were able to manage it as a chronic infection. The experience of HIV as a chronic infection is dependent upon contingent factors that affect access to treatment [14–19]. But even when it is managed as a chronic illness, HIV care presents its own challenges. This article discusses present-day clinical ethics issues in HIV care in Canada. In the context of a larger project examining the shift in HIV care from specialist to primary care environments, we asked people living with HIV as well as their health care providers about the quality of their care, successes, difficulties, and sources of conflict. The objective of our study was to investigate and document clinical ethics issues experienced in HIV care.

Recent clinical ethics literature on HIV addresses a variety of themes. Among these are the criminalization of failing to disclose HIV positive status to sexual partners [20–25]; the recent push to treat HIV with ART as a way of preventing transmission, known as “treatment as prevention”, which can cause a conflict between duties to the patient versus public health priorities [26–31]; and adherence^1^ to ART among members of vulnerable groups [32–36]. In our study we gave research participants opportunities to discuss these ethical issues in particular, as well as any others they may have experienced. We therefore sought to determine whether some of the more prominently discussed ethical issues in HIV care are actually occurring in the HIV clinics we studied, and to determine what other ethical issues might affect day-to-day care.

## Methods

### Institutional ethnography

This study utilized an institutional ethnography (IE) methodology. IE is a method for studying the social relations that shape and organize everyday experience [37–40]. The focus in IE is not so much on the individual participant in the study as it is on “the social” itself, understood as the ongoing structural and social determinants of everyday activity [35]. These determinants include institutional work processes – the implicit norms, and explicit rules of daily work that organize activity. In our study, the “work” in question is defined broadly to include both the work of health care providers involved in the clinical and social care of people living with HIV, as well the health work contributed by people living with HIV themselves devoted to their own care [35]. The IE researcher Liza McCoy states that “[t]he health care of a person living with HIV involves a complex, daily work process that loops from the home and everyday spaces of the individual into the sites of professional medical service and back again” [34].

We investigated the work performed by both groups (health care providers and clinic clients^2^) – work that involves managing, responding to, and occasionally provoking ethical issues in clinical care for HIV. IE exposes the institutional orders, power imbalances, and social factors at play in coordinating this work. The institutional order in which work is performed often consists of activities directed by textual materials [41]. In our study these influential texts included Supreme Court of Canada rulings, provincial government legislation, professional guidelines, and published research findings. IE is a method that attends to these texts and traces out their influence on everyday activities. The relations instituted by such texts are “translocal” in nature – they extend across local contexts and standardize activities in multiple settings. Everyday processes of treating HIV in a clinic and of managing one’s health are implicated in organizational effects that originate from beyond the everyday setting. The goal of IE in this study was thus to assemble the health work experiences of those involved with ethical issues in HIV care, and to display how these experiences are coordinated – socially, locally, and translocally [41].

### Description of our study

Our institutional ethnography researcher worked in the field for one year at three different HIV clinics – one in the Canadian province of Newfoundland and Labrador (NL), and two in the province of Manitoba (MB). The clinic in Newfoundland and Labrador is based in the city of St. John’s, which is a small urban centre with a population of about 200 000 people. The St. John’s clinic is the only HIV clinic in the province. Both clinics in Manitoba are based in the capital city of Winnipeg, which is a mid-sized urban centre of just under 700 000 people. All clinics in the study serve a mix of urban, suburban, and rural residents. The geographic diversity of the populations served by the clinics in our study was a desirable feature, since we sought to capture ethical issues affecting people living with HIV both within and outside of urban areas. Each clinic in our study also has substantial numbers of female patients – in Newfoundland and Labrador these were often women living in nonurban areas, and in Manitoba many participants were Indigenous women.

Our researcher observed and interacted with health care providers and clinic clients over a period of 5–6 months in each clinic. She documented clinical interactions between providers and clients, conducted interviews with members of both groups, examined medical records, and analyzed textual materials. The researcher read through medical chart notes, copies of correspondence between physicians and patients, and between primary care providers and specialists. She studied a large number of documents such as clinic and professional policies on the use of ART, therapeutic guidelines for the treatment of HIV in primary care, guidelines on the management of co-infection, job descriptions, communicable disease surveillance reports, the results of internal quality-improvement initiatives such as a patient satisfaction survey, and other relevant materials.

The interviews were semi-structured in nature, with opportunity for the researcher to probe topics further and pose follow-up questions. We interviewed a total of 22 people living with HIV, and 20 health care providers, including primary care physicians, infectious disease specialist physicians, clinic nurses, nurse practitioners, clinic-based and community-based social workers, pharmacists, and others. We sought to include any health care worker involved in the health or social care of people living with HIV who attended one of the clinics in our study.

Amongst the clinic clients living with HIV, our researcher interviewed and interacted with people from many social locations – including gay, bisexual, and heterosexual men, men and women living in rural areas and urban centres, Indigenous^3^ men and women, people with a history of injection drug use, affluent people and people living below the poverty level including people who are homeless, and people with physical disabilities. We included any adult living with HIV who attended the clinics in our study, and only excluded potential participants on the basis of inability to consent to participation. We sampled purposively in order to introduce as much diversity as possible in the group of people living with HIV included in our study. Clinic clients were contacted in various ways to request their participation. Some volunteered after seeing advertisements for our study in clinic waiting areas, or after hearing about the study through word-of-mouth. In other cases, health care providers told clients about the study during clinic visits and were directed to our researcher to discuss participation.

During interviews with clinic clients our researcher asked general questions about what is going well with their HIV care, what could be improved, what changes might have occurred in the management of their HIV over time, and about how much they feel involved in decisions about their care. We also asked targeted questions about how they manage disclosing their HIV positive status with family, friends, and intimates, as well as questions about any barriers they experience with adherence to ART, and how their health care providers interact with them on adherence issues. With such questions we sought to uncover any ethical issues clinic clients had experienced.

Our researcher asked health care providers general questions about challenges they face in providing clinical care for people living with HIV, as well as questions about what ideal care would look like. She asked for their thoughts on the “treatment as prevention” paradigm. The interviews with health care providers also included questions about how they work with clinic clients on the issue of disclosing their HIV positive status to sexual partners, about how they manage legal issues in their work, and about challenges with adherence to ART. In this way, the interview questions included a mix of general probes meant to uncover ethical issues, as well as specific inquiries about issues that are prominent in recent ethics literature on the clinical care of people living with HIV.

Our researcher compiled participant observation notes during clinic interactions between health care providers and clients, and between care team members themselves. She observed patient appointments with care teams and with individual health care providers in which care plans were discussed. Each appointment lasted about 45 min. The researcher also participated in formal and informal meetings between health care providers of varying duration. She attended to the ways in which clinic clients were involved in decision-making, the dynamics of communication, and any instances of difficulty or misunderstanding experienced by clinic clients or their health care providers. Similarly, in her review of patient records, the researcher noted how professionals communicated with each other through letters or charts, and any care challenges documented in this medium. These sources of information were used to examine and trace out the relations between professionals, and between health care providers and clinic clients.

All interviews were audio recorded and transcribed. The transcripts along with field notes based on clinic observations and on patient medical records were made available to the data analysis team. This team consisted of a multidisciplinary group including the ethnographic researcher herself, and others with expertise in clinical ethics, institutional ethnography, qualitative research, health services research, and the clinical care of people living with HIV and of marginalized populations. We embedded verification of our results into the study design through the constitution of our analysis team as an intersubjective multi-expert group [42]. Each member of the analysis team read through transcripts, observations notes, and relevant documents independently. We convened a series of meetings over several months to discuss the ethical issues that we each found being discussed or depicted in the data. Through the analysis of data, we identified a series of clinical ethics issues in HIV care experienced by study participants. Each team member compiled a descriptive list of ethical issues they found in the data. During our meetings, and in the drafting of this article we arrived at a discursive consensus about the ethical issues that emerged in our study, and about the important social and institutional factors contributing to these issues. We sought to catalogue these issues and to trace out the relations (social, textual, local, translocal) that play a role in making them ethical issues. Data “saturation” was evidenced by the thematic coherence across our sources of data that we found upon analysis.

The concept of “ethical issue” in the clinical setting can be interpreted variably. We encouraged our study participants to discuss any issue they saw as ethical in nature, in order to avoid excluding any issues that could possibly be relevant. However, in our analysis we have included, amongst the clinical ethics issues in our findings, those issues that health care providers and clinic clients devote work processes to resolving. These issues can often be described as dilemmas between the classical ethical principles of respecting autonomy, beneficence, nonmaleficence, and justice. Our goal was to describe the clinical ethics issues we encountered in the data and the work processes that research participants devoted to the management and resolution of these issues. We acknowledge that ethical issues in the clinical setting have their roots in, and are complexly entwined with, poverty, racism, oppression, and other problems of social and institutional structures faced by clinic clients and care providers. These social problems are not always easy to describe using the language of clinical ethics principles. Though we have not described the social determinants of health and well-being as themselves clinical ethics issues, we account for these determinants as causes and contributing factors to such issues.

## Results

As we have discussed, there were a number of ethical issues about which we had pre-planned questions in our interview guides. There were also other ethical issues that emerged out of general questions about quality of care, communications, and the like. We organize our discussion of results with the former type of ethical issues presented first, and with the latter type of ethical issue following.

### Treatment as prevention versus respect for autonomy

The treatment as prevention paradigm is premised on the idea that using ART in the treatment of people who are newly diagnosed with HIV as soon as possible, reducing their viral load, and maintaining viral suppression, will also reduce the likelihood that the diagnosed person will transmit HIV to others [43]. Viral suppression reduces transmissibility [44]. Over time, the reduction of the number of people within the population who are infectious could effectively stop HIV from spreading. Combined with an expansion of testing and identification of people living with HIV, the treatment as prevention paradigm could be an effective way of controlling HIV within a population.

From a clinician’s point of view, the public health interest in controlling the spread of HIV within the population at large through the treatment as prevention strategy could set up a conflict between a clinician’s duty to the client, and his or her duty to the health of the public [31]. On the one hand, there is a duty to respect a client’s informed consent about initiating or refusing treatment, such as ART. On the other hand, a client’s refusal might run counter to the public health interest in reducing the viral load of all clients as a way of reducing the likelihood of transmission. Pushing treatment as prevention on a patient in the interests of public health could thus violate the client’s informed consent [28]. This dynamic could create a dilemma between the obligation to respect the autonomous refusal of care versus a health care provider’s duty of beneficence towards the health of the public.

In our study, we asked health care providers their thoughts about treatment as prevention. In general, health care providers reported that they defer to the client’s informed consent. They recommend initiation of ART based on clinical guidelines, but also respect refusals of treatment. Consider the following exchange

Health care provider (HCP): … the guidelines now are saying you just offer it…

Interviewer (I): And that’s the guidelines. And there’s nothing you can do.

HCP: Yeah, it’s up to them [the clients] … Most people choose to take them [ART] … Because the literature now is that the sooner people start the better their immune system is…

I: Right. So this idea that even if you don’t need it right now to boost your immune system you may have some protective effects because of it.

HCP: Yeah. So we didn’t look at it as a community health thing. We look at it from the patient’s perspective.

I: And if they’re not interested in going on treatment …

HCP: That’s up to them. (health care provider, NL)

The health care providers involved in our study tended to see the benefits of ART for preventing transmission. Even more importantly, however, they were comfortable recommending the initiation of ART because of perceived benefits to the client. Thus, health care providers who participated in our study reported that they give priority to the client’s informed consent over the public health benefits of treatment as prevention.

Prior to the development of guidelines supporting the early initiation of ART, there was no professional consensus in favour of this practice [45]. In the exchange quoted above, the health care provider suggested that the norms underlying this approach to treatment arose from published research demonstrating the benefit of early initiation of ART, and from subsequent changes in the treatment guidelines. There is also a strong medico-legal injunction underlying virtually all clinical encounters that informed treatment refusals must be respected.

### Criminalization of HIV nondisclosure

In 1998 the Supreme Court of Canada set a precedent for the conditions under which a person who is HIV positive can be found guilty of a crime, such as assault, as a result of failing to disclose his or her HIV positive status to a sexual partner. This ruling (*R. v. Cuerrier*) stated that such a person is guilty if he or she was dishonest about being HIV positive through lying or failing to disclose, and if this dishonesty resulted in “significant risk of serious bodily harm” through sexual contact [22, 46].

In 2012 further Supreme Court of Canada rulings in *R. v. Mabior*/*R. v. D.C.* clarified the previous precedent. In these cases the Supreme Court specified that if the person has a low viral load and uses a condom, then there is not a realistic possibility of transmission and no significant risk of serious bodily harm, so failing to disclose that one is HIV positive is not a criminal act [47, 48]. However, a low viral load, but with a failure to use a condom, and a failure to disclose that one is HIV positive, was deemed a significant risk of serious bodily harm, and was therefore judged a criminal act. This judicial interpretation of the level of risk runs contrary to current scientific evidence and expert opinion [49]. These rulings suggest that even condom use is not enough to avoid prosecution for nondisclosure. One must also have a low viral load to avoid criminal charges for failing to disclose, though what constitutes “low” viral load was not defined by the court.

These rulings have clinical ethics implications for people living with HIV, and for their health care providers. If a clinic client is arrested or charged with a crime because of nondisclosure, medical records could become subject to subpoena, and health care providers could become witnesses or could be called to testify in a criminal proceeding. These possibilities could influence the counselling practices of health care providers and the experiences of clinic clients [25]. The Supreme Court rulings could determine the information health care providers impart to clients, and the introduction of legalities into the clinical relationship could influence the level of trust and the quality of the information that is shared. These legal rules could affect a health care provider’s ability to comply with the ethical duty of nonmaleficence. Furthermore, factors such as Supreme Court rulings, counselling practices, and levels of trust, have the potential to affect the intimate sexual practices of people living with HIV, their feelings of fear, self-worth, and safety.

We asked clinic clients in interviews about their disclosure practices, and about any legal concerns they had related to their HIV status. We also asked health care providers about legal issues they had encountered. Both groups of participants brought up the criminalization of nondisclosure in response to these questions. Criminal cases involving nondisclosure were familiar to many research participants in both groups. In Newfoundland in 1993, a man named Raymond Mercer had been found guilty of endangering the lives, safety, or health of the public because he transmitted HIV to two women via unprotected sex [50]. Mercer had failed to disclose that he was HIV positive to these women. He was sentenced to eleven years and three months in jail [50]. When we asked research participants in Newfoundland about their awareness of the nondisclosure issue, several health care workers and clinic clients mentioned the Mercer case.

In general, the clinic clients living with HIV we interviewed stated that they have a personal policy of disclosing their HIV status to all sexual partners. The disclosure process can happen in various ways – verbally of course, but also through information provided on internet dating sites, or through apps designed to help people find sexual partners. One clinic client described his disclosure practices in this way:

There’s one that’s actually pretty exclusive for HIV: barebackRT.com … in the biography the little bio it will say “positive don’t care”, “negative” then you’ll say who you look for, like you look for negative only, positive only … That one kind of keeps you out of trouble if you want to just hook up with positive guys or undetectable – whatever you’re looking for. If you’re on Grindr, Grindr is not bad but actually it tells you what your little tribes are and you can put whatever type of people: bears, twigs and you can even put positive in there too you know. (clinic client, MB)

As this research participant pointed out, disclosure practices are often embedded in, and are mediated through technology. The Supreme Court rulings, of which virtually all research participants seemed to be aware, play a role in organizing these practices toward a fairly standard position of disclosing to sexual partners. Other studies have discussed the difficulties associated with disclosing that one is HIV-positive [22], though the participants in our study tended not to highlight these difficulties in their own experiences.

From the perspective of health care providers, many noted that their work practices involve counseling clinic clients about the law regarding disclosure, and that they also make an effort to involve spouses or other partners in the counselling process, encouraging clinic clients to bring them to appointments. One provider described her procedure: “We chart that we’ve made sure we told them the disclosure laws. We chart that the first time we see them to make sure that you know it’s documented or whether or not they brought a partner in with them” (health care provider, MB). Potential legal or ethical issues that might arise as a result of nondisclosure are managed through counselling early in the clinical relationship, involving other potentially affected parties, and through textual documentation on the client’s chart.

These attempts to manage the possibility of nondisclosure themselves might have untoward effects. One clinic client said that he was annoyed about being lectured by his family doctor to disclose to every sexual partner, even though his viral load was undetectable and he always used condoms. The doctor mistakenly informed this client that he had a higher standard of responsibility (“always disclose”) than what is required by the Supreme Court of Canada. As discussed above, the Supreme Court rulings state that disclosure is not required when a person living with HIV uses a condom and has a low viral load. In this way the legal implications of HIV non-disclosure can invade the client-provider relationship, impact the quality of these relations, and confuse practical advice regarding the prevention of HIV transmission.

Furthermore, sometimes nondisclosure occurs – another potential untoward outcome. People fail to disclose for all sorts of reasons, and counselling may be ineffective in preventing this from happening. A health care provider described such a scenario:

the biggest [problem] would be probably to diagnose somebody knowing that they got it from somebody that I know. That was the biggest that I had to deal with when, for example, you know the person, they’re not on medication … They’re refusing. You tell them to use condoms, they say they do but they don’t, and then they end up with a boyfriend, and then the boyfriend gets tested and they’re positive, and they didn’t tell the boyfriend. So then you know the background and the details but you can’t say it out loud to the other person. (health care provider, MB)

On the one hand, the health care provider had a duty to protect a client’s privacy regarding the client’s HIV status. On the other hand, the health care provider had information about probable harm to another person on the part of the client who appeared to have transmitted the virus to a boyfriend. But sharing this information with the boyfriend would be a striking breach of confidentiality, and the legal implications of HIV nondisclosure increase the potential for harms to the client. Nonetheless, ongoing nondisclosure by the client could result in harm to others. The health care provider managed the ethical difficulty posed by a client’s failure to disclose by maintaining the client’s confidentiality. Other health care providers described physicians and nurses in their clinic becoming involved in court proceedings over nondisclosure, and another recounted being contacted by police about a suspected nondisclosure case and refusing to provide information without being given a warrant.

We have presented a number of ways in which nondisclosure is managed in the clinic – for example through counselling, or protecting confidentiality when this is legally possible. One further method for managing the nondisclosure issue is through documentation. However, this method can have unintended effects. A health care provider outlined a way criminalization might encourage the spread of HIV:

I think people have put off, you know, getting tested though because they do have very risky unprotected sex and I think they put off being tested so that it’s not on paper that they’re diagnosed so they can continue their behaviour. (health care provider, MB)

In this case, the consequences resulting from being documented as HIV positive were understood as a deterrent to testing and accessing treatment. This health care provider believed that some people might be afraid of being denoted HIV positive “on paper” because such a notation could force a change in behaviour. Criminalization could impede the realization of public health goals if HIV is spread because of this fear.

In summary, clinic clients involved in our study described a personal policy of disclosure as a way of preventing legal problems related to nondisclosure. Health care providers managed the nondisclosure issue through counselling and documentation.

### Adherence to treatment

One of the chief obligations of health care workers to their patients and clients is to provide benefit – otherwise known as the duty of “beneficence”. Care providers might believe that they have failed in this ethical duty if the treatment they offer is not successful. But success in the treatment of HIV often depends on adherence to ART – which requires the participation of both provider and client in the health work of HIV care [35]. Sometimes, for various reasons, people do not successfully adhere to treatment.

[T]he people … I have the most consistent interaction with are actually the folks who are nonadherent. So they surface, you know, and I do all kinds of stuff with them to get their medications again and see them and talk to them about adherence – not about adherence but about their lives to see what we can work out together – and then they’ll disappear for a while … I used to have more of them but a lot of those folks over time actually died. (health care provider, NL)

Many participants, clinic clients and health care providers alike, gave accounts of the difficult, ongoing, participatory health work of maintaining adherence. Many described the hard work of maintaining trust and problem solving around side effects, housing issues, addictions, education about HIV and how ART works, and a myriad of other factors that affect adherence. It may be difficult to accept that you have fulfilled your ethical duties of beneficence when these efforts are for nought and your clients die.

Nonetheless, all of the evidence in our data suggests that the health care workers who described these efforts are highly beneficent. All health care providers in our study tended to adopt a policy of trust and deference toward the personal choices of clients, even if this policy resulted in nonadherence. They tended to be noncritical, and to approach the issue from a constructive problem-solving perspective, while continuing to encourage adherence.

In the clinical context, one way of managing the issue of adherence is through monitoring and questioning the client. At every appointment, health care providers asked clients about whether they had missed their medications, and followed up if blood work indicated less than adequate adherence to ART. The development of resistance to different treatment regimens often requires changes or adjustment to medication, so keeping watch over adherence is a necessary clinical practice. Nonetheless, some clinic clients expressed annoyance about this questioning. One client described the dynamic in the following way:

It has everything to do with stigma … like it is my job to be a good, behaving, positive person always going for blood work, always adhering to their medication because I hesitate to tell my doctor if I sometimes miss my meds. You know I hesitate to tell them like it’s a pain in the butt that I have to alter my life so I can come in for blood work once every five or six months. (clinic client, MB)

This clinic client linked the questioning about adherence with HIV stigma. The participatory nature of health work can produce tension if there is an implicit assumption that people living with HIV must adhere to treatment – or “behave”. It may be difficult to maintain trust in such circumstances. While the health care providers in our study maintained an attitude of respectful problem-solving in the management of adherence, these interactions can still exhibit ethical tension.

### Protecting confidentiality

We have already touched on the protection of confidentiality as a way of dealing with nondisclosure situations in the clinic. The topic of confidentiality also emerged in our study as an ethical issue in two other important ways. Unlike the ethical issues discussed earlier, we had no specific questions in our interview guides that prompted participants to discuss confidentiality.

The first example relates exclusively to the clinic in Newfoundland. This clinic had features in its built environment and policies for scheduling appointments that were designed to ensure that patients coming for visits did not come into contact with each other. Clinic appointments were spaced out one client at a time, and waiting areas were organized so that waiting clients would not be able to see other clients leaving, and would not come into contact with other people who were in the building for other reasons. According to one health care provider, these measures were put into place at the request of a number of clients who were unhappy with a previous arrangement in which they had to wait for appointments in a common area with clients for other clinics: “they didn’t like being set in the room because they felt all eyes were on them, that they knew that’s why they were there” (health care provider, NL). Even with the new arrangement, some clients went to great lengths to avoid contact. A health care provider described one particular client as:

one of these ones who sneaks in the back door, calls me when he’s outside, “All right, I’m on my way up just be waiting for me, make sure there’s nobody there,” won’t get his blood work done [in the lab] and he’ll come up here [to the clinic] and get his blood work done, won’t go anywhere else. He’s one who I’ve actually had to go to his home and drew blood work because he didn’t want to come in. (health care provider, NL)

Part of the explanation for this concern around maintaining privacy is that Newfoundland and Labrador has a small population (only about 500 000 people), and it is relatively likely for a client to see someone he or she might know while waiting for an appointment – “somebody knows somebody who knows somebody, you know” (health care provider, NL). The clinic in St. John’s served people who live in rural areas, along with those in urban and suburban areas of the city, and the experience of lacking privacy becomes magnified in small-town or rural Newfoundland [51].

From one perspective, maintaining confidentiality by sheltering HIV clinic clients from unwanted contact can be seen as culturally competent care. Within a cultural context with a heightened degree of HIV-stigma, homophobia, or other intersecting biases, the protection of confidentiality can be seen as an ethical virtue. However, in a clinic with a diverse population of clients, not all have the same values or culture. Some research participants saw the measures used to protect confidentiality as “perpetuating the stigma” (clinic client, NL). According to this reasoning, there is fear about being known as HIV positive only because HIV is a stigmatized illness. Thus, excessive privacy measures only accommodate stigma, when stigma should instead be challenged with openness or normalization. One research participant argued that there was a downside to privacy measures that “push the stigma”. He speculated that, “There are probably another 500 cases in Newfoundland that haven’t been detected because of that” (clinic client, NL). In his view, a reluctance to get tested for HIV results from the perpetuation of HIV-stigma.

This disagreement between the values of clinic clients created a dilemma for health care providers and their managers. Satisfying the request for enhanced protections for confidentiality means, at the same time, being unable to satisfy those who want more openness as a challenge to stigma, or to enable community connections to form in the clinic setting. Clinic workers thus face a dilemma between the duty to protect confidentiality versus a version of the duty of beneficence that includes confronting stigma. The health care workers in our study were aware of the issue, and they managed the dilemma by deferring to the clients who prefer greater anonymity – who do not want to be circumstantially identified as living with HIV.

Our second example of confidentiality as an ethical issue in our study likewise involved life in rural areas. The clinics in Manitoba also serve people living in rural areas of the province – small towns and Indigenous communities. Confidentiality can be easily compromised when medications are shipped to nursing stations, where all health care is coordinated in these isolated and rural areas. Several health care providers noted that nursing stations often have community members on staff or hanging around who might be able to see medications being delivered. The fear of HIV is so great in some communities that people have been ostracized because of their HIV status. A health care provider noted that, “it happens. We’ve had people that have to endure terrible situations in their home community and be isolated and leave. They leave” (health care provider, MB). Similar problems arose around the use of tele-health facilities in nursing stations where it is difficult to maintain privacy during tele-consults.

A research participant described some measures for maintaining client confidentiality in the shipping of medication. She said that:

pharmacies, if they know that there’s issues of confidentiality, they’ll do their best to be discreet about putting them in a bag, you know, making sure that the bag isn’t labeled out on the patient name, and then … some have vans that will deliver them to the patient. So there are ways to work around it. (health care provider, MB)

Clinic personnel including pharmacists have developed work processes designed to avoid the textual identifier on a bag of medication that is a danger to the client. The centralization of care in an urban centre away from one’s home community, and HIV-related stigma can create threats to maintaining confidentiality when care relationships extend from the urban centre to the rural area.

### Access to medication

Canada has a universal, single-payer, health care system for physician and hospital services. However, publicly-funded health care does not extend to pharmaceutical coverage outside of hospitals. Throughout the country there is a great variety of Federal and Provincial government programs, and private insurance plans that offer some degree of prescription drug coverage, often tied to employment or membership in certain groups. However, there is no guarantee for citizens of either Manitoba or Newfoundland and Labrador that one of these drug plans will cover the total costs of ART. For those who do not have 100% coverage through a private, Federal, or provincial plan, each of these provinces offers a “catastrophic” drug plan as a kind of last resort. These provincial plans require payment of an annual deductible on a sliding scale according to income. However, the deductible can be in the thousands of dollars for people with even modest incomes of $30 000 or $40 000 a year [52, 53]. There are further administrative requirements for applying to these drug plans that can be an impediment or delay to access, such as the requirement that applicants must provide information about their income from annual tax returns – documentation that many people may not have. Our interview guides did not contain specific questions about financial or administrative barriers to ART access, but this issue emerged as a prominent concern in our interviews and other sources of data [54].

Because of the deductible, many people living with HIV simply cannot afford ART. One clinic involved in our study maintained a list of “at least 30” people who were not receiving ART because they could not manage the cost of the deductible (health care provider, MB). Considering that without treatment HIV is a life-threatening disease, this is a striking number of people for whom cost is a barrier. Clinic clients recounted stories of people who had delayed treatment with ART because they had not filed tax returns for a number of years, and thus could not apply for provincial drug coverage. One participant described the horror he felt when he was told his annual deductible would be $6000. Even when people living with HIV can pay the deductible and access ART, the cost can have an effect on their quality of life. In the clinical context, poor coverage for ART makes it difficult for health care providers to fulfill their duty of beneficence.

Health care providers and clinic clients outlined the extensive participatory health work involved in getting access to ART. One participant pointed out that, “everyone needs a social worker when they go through that, talking about our different programs, what’s your medication coverage” (health care provider, MB). The details, requirements for drug coverage, and paperwork can be quite complicated. A clinic client shared an anecdote about the efforts his care team directed at resolving a dispute between a provincial drug program and his private insurance:

So there’s a drug in my regime that is covered by [the provincial plan] but only if the original [private insurance] provider will not cover it. So when [the private insurance plan] learned that, they refused to cover the drug for me, and then [the provincial plan] refused to cover the drug for me as well too, and I think the overall cost is like $1500 a month or something along those lines. So when that happened and I was denied, they [the health care team] contacted [the private insurance plan] directly for me and fought them. So I was in the office when they were on the phone for a good like two-and-a-half hours arguing with these guys and then got it for me. (clinic client, NL)

In this story, securing access to a life-saving drug required a startling degree of advocacy to break the impasse. Without such advocacy, an administrative barrier can place the client’s health or financial well-being at risk.

## Discussion

### Similar studies

There is a wide variety of studies using IE and related methodologies to research the health care experiences of people living with HIV. These studies usually lack a focus on clinical ethics issues, or tended to focus on only one of the issues identified in our study. We have found no IE studies whose purpose was the identification of clinical ethics issues in HIV care, or the management of such issues.

Several studies have used IE to examine clinical relationships in HIV care [34], the criminalization of HIV nondisclosure [22], and the health-work of adherence from the standpoint of people living with HIV [32, 35, 36, 55, 56]. Other studies have examined HIV care in other countries using ethnographic methodologies or related qualitative methodologies [57–61] though these studies do not tend to focus on issues in clinical ethics. One exception is a study of moral distress among Ugandan nurses who treat patients with HIV that used a critical ethnographic method [62]. A number of Canadian studies used qualitative methodologies to examine the health care experiences of people living with HIV [63–65]. One such article examining perinatal care in Canada for people living with HIV identified the disregard of confidentiality as a negative care experience [64]. Two recent Canadian studies used qualitative methodologies examine health care providers’ experiences and perceptions of adherence to ART [66, 67].

### The social determinants of clinical ethics issues

The issues of treatment as prevention [20–25], the criminalization of nondisclosure [26–31], and ART adherence [32–36] have been extensively discussed in recent ethics and social science literature. In addition to these, our study found that issues of confidentiality, and access to ART are part of the everyday work of clinic clients and health care providers in HIV clinics. As discussed in the previous section, each of these ethical issues can manifest in the form of clinical dilemmas in which ethical duties come into conflict with one another. For example, health care providers have duties to their clients that might conflict with duties towards the health of the public. The protection of confidentiality might conflict with efforts to reduce stigma. These clinical dilemmas often have distal causes outside of the clinic. The issue of access to ART results from social policy decisions made by provincial governments and other ruling authorities beyond the clinic, yet the effects of these decisions must be managed within the clinic. Furthermore, the social determinants of poor health and well-being experienced by some people living with HIV were often contributing factors to ethical issues in the HIV clinics.

The most overwhelming and lasting impression resulting from our study is that for many people living with HIV, their social circumstances are a profound threat to their health and well-being. Many research participants documented instances of violence, racism, substance use, poverty, homelessness, intergenerational trauma, HIV stigma, homophobia, among other threats to health and well-being. The most affected were clinic clients who were homeless and experienced multiple forms of structural disadvantage. As an example, a health care provider described a client of hers:

she’s very complicated, she’s a solvent user, she’s Aboriginal, very, very, very traumatized person, street entrenched, sex worker, just lots of issues, injection drug user. So she was not accessing care at all in any consistent way for any reason. (health care provider, MB, referring to clinic client)

Several clinic clients recounted stories of violence tied to intergenerational trauma, colonization, and further cycles of poverty, violence, and problematic substance use. One said “my birth mother was constantly feeding me the alcohol so I had no break” (clinic client, MB); another “was with his birth mother when she was murdered or committed suicide” (family member, MB referring to clinic client). Out of three children the mother left behind he “demanded the most attention screaming as a drug baby [because] his mother was on cocaine and heroin” (family member, MB referring to clinic client).

In the health care context, some of our research participants reported being mistreated or being denied care by health care providers because of their marginality. In one example, a physician attempted to coerce a woman into having an abortion when she was diagnosed as HIV positive. Another participant was denied care in an emergency department when she had a facial fracture, and suspected that the physician was motivated by racism, anti-HIV bias, disapproval of her substance use, or all three factors. None of the instances of mistreatment we heard about in our study related to care in any of the HIV clinics in our study, but instead occurred in other health care environments.

Social determinants of poor health and well-being can contribute to becoming HIV positive, and can create difficulties with maintaining care for HIV [68–71]. Data from client health records in our study indicate that factors such as being homeless and substance use correspond with problems adhering to ART, and challenges with attending clinic appointments. Protecting confidentiality is identified as a challenge and a necessity, in part, because of HIV stigma. Access to medication is an ethical issue because poverty, homelessness, and associated factors can put ART out of reach. The criminalization of nondisclosure, and treatment as prevention become complicated issues when clinic clients have challenging everyday lives, and experience structural disadvantage. Life circumstances such as experiencing violence, trauma, or problematic substance use issues are detrimental to health and well-being even when not living with HIV. Social disparities can contribute to the spread of HIV, and vice versa – a phenomenon known as the theory of “syndemics” [70, 72].

The clinic clients in our study who were among the most disadvantaged expressed a sense that mortality was near. One client who was homeless said “I’m already freaking dead anyway. You know what I mean? Kind of like the walking dead” (clinic client, MB). When our researcher offered to share the results of the study with her at a future date, the participant said, “if I’m alive in a year like I don’t know right”. Another participant wondered “like well how much time do I have left? Do I have enough time to prepare for anybody to know like what’s going to happen?” (clinic client, MB). A further research participant said that he had been “noticing more and more people who are HIV turning to crystal meth and it’s usually only a couple of years they’re alive you know” (clinic client, MB).

The health care providers in our study were aware of these issues, and were constantly trying to help clients with them. One participant who worked with especially vulnerable clients explained that,

most of them struggle with future orientation … it’s like they have a different priority. They live day to day: they don’t care about what’s happening tomorrow or in a couple of hours … like taking medications to prevent them from getting sick in the future doesn’t register … life is hard enough that they don’t really care about HIV. (health care provider, MB)

When HIV is just one among many threats in one’s life, and when there are more imminent dangers, seeking and adhering to treatment for HIV may compete with more immediate priorities, or may not be a priority. The social context of living with HIV is often complex and, for people living with multiple extreme social disadvantages, their perspective on the work of managing HIV may be different from the perspective of health care providers. The ability to work towards future goals of health and well-being may become compromised if it is hard to believe that one will live very far into the future. For some people living with HIV who face such danger in their lives, accessing care and adherence to ART may have less importance than day-to-day survival. The fact that they live in such peril is itself a significant ethical issue.

Many of the ethical issues we have examined have their roots in translocal relations in which an external authoritative text organizes behaviour in the clinic or the local setting. For example, provincial government drug programs – effected through legislation and regulations – limit access to ART by requiring deductibles or administrative requirements that cannot be met by some people living with HIV. Supreme Court rulings influence clinical behaviour and sexual behaviour by criminalizing nondisclosure of HIV status. Nonetheless, though many ethical issues are subtended by such translocal ruling relations, we should not lose sight of the highly localized nature of these issues. Local realities contribute to the institutional order in which these ethical issues occur. For example, some ethical issues we have discussed are especially prominent within certain populations. Racism and colonialism directed at Indigenous peoples can be a barrier to care that is not experienced by other groups. Problematic substance use can be a uniquely challenging determinant of adherence to ART. Furthermore, some ethical issues arise out of the local history of HIV. Heterosexual people living with HIV in rural Newfoundland may have values and expectations of care that are different from the values and expectations of urban gay men. Such a conflict of values could be a source of the dilemma about how to manage issues of confidentiality in the clinic. The organization of care at nursing stations in remote Indigenous communities also gives rise to efforts to protect confidentiality, as well as conflicts arising from the colonial system of health care delivery. The experience of living with HIV is not uniform across Canada. As such, we can expect that different local settings will be faced with their own ethical issues.

### Implications for practice

Health care providers and people living with HIV are highly aware of the clinical ethics issues they face in HIV care, and have developed norms and processes for managing the issues that arise.

- In the case of potential conflicts that arise out of “treatment as prevention” efforts, health care providers defer towards accepting the client’s informed decision-making.
- Health care providers manage difficulties related to the criminalization of nondisclosure through counselling, documentation, and the protection of confidentiality whenever possible. Clinic clients tend to have adopted a norm of disclosure.
- Nonadherence is managed through perseverance and a nonjudgmental approach.
- In situations in which confidentiality is at risk, the clinics in our study have enacted procedures that minimize the risk of breaches of confidentiality. Conflicts between confidentiality and other values, such as stigma reduction, are more contentious, but it appears that the clinics have sought to preserve confidentiality rather than endorsing another approach.
- Issues of access to HIV medications are managed through determined advocacy by and on behalf of clinic clients.

These findings do not translate into directives about how to resolve ethical issues in HIV clinics. In a particular situation with circumstances that might be unique, a more thorough normative analysis would be required in order to determine whether a proposed resolution is ethically justified. Nonetheless, our findings suggest a few possible procedures and some common approaches taken by people living with HIV and their health care providers for resolving some common ethical issues encountered in HIV clinics in Canada.

The negative social determinants of health and well-being that appear to be at the root of many clinical ethics issues cannot be fully addressed or resolved in the clinic and are translocal in nature. Improvements in the lives of people living with HIV so that these issues are addressed at their source will be a highly complex task, requiring political change, structural interventions, and a more just society.

### Limitations

We cannot claim to provide a comprehensive account of ethical issues in HIV care in Canada. The clinics we studied are not representative of the Canadian population. In particular, larger urban centres such as Toronto, Montreal, or Vancouver were not represented in our study. The fact that there is less ability for people to choose between HIV clinics in small centres such as St. John’s may have affected the ethical issues found in our study of these centres. The clinics involved in our study also tend not to be directed at serving specific populations, such as gay or bisexual men, for example. The ethical issues in such population-specific clinics may be different from what we have found in our study.

We attempted to capture a diversity of people living with HIV among participants in our study. However, we were unsuccessful in our attempts to include refugees or migrants from areas in which HIV is endemic, or who had been living with HIV prior to arrival in Canada.

A group of community scholars living with HIV provided feedback on our research materials such as study design, data access plans, and interview guides. However, this group was small and could not, in any case, represent the diversity of people living with HIV. We have attempted to reduce the impact of observer bias by enabling a multi-disciplinary group to conduct the data analysis, through the use of textual materials available to all members of the analytical team, and verbatim transcripts of interviews for analysis. Nonetheless, the authorship group does not include anyone with an Indigenous background, anyone living with HIV, or representation from non-white ethnospecific groups. These demographic factors may have influenced our findings or analysis.

## Conclusions

As has been discussed, we found evidence that people living with HIV and their health care providers were managing ethical issues in clinical care related to the phenomenon known as treatment as prevention, the criminalization of nondisclosure of HIV status, and adherence to ART. We also found that both groups deal with confidentiality issues, and problems with access to ART. Several people living with HIV involved in our study who experienced the most acute structural disadvantages expressed an awareness of their imminent death because of the dangerous circumstances of their lives. Clinical ethics issues in HIV care occur within the context of these negative social determinants, and this context arises from outside of the clinic as part of the everyday experiences of people living with HIV. Clinical interactions, including the management of ethical issues, form part of the everyday experience of poverty and social inequality.

## Abbreviations

AIDS: Acquired immune deficiency syndrome
ART: Anti-retroviral therapy
HCP: Health care provider
HIV: Human immunodeficiency virus
I: Interviewer
IE: Institutional ethnography
NL: Newfoundland and Labrador
MB: Manitoba

## Footnotes

1 By “adherence” we mean “taking medication/undergoing recommended treatment”. Some authors do not use the term “adherence”, while others do. While the term “adherence” is an improvement over the term “compliance” – which implies that people are complying with orders or directives when undergoing treatment – we acknowledge that some might not fully endorse the term “adherence” either.

2 Throughout the article, we refer to people who are living with HIV and accessing clinic care as “clinic clients” for the purposes of brevity. We acknowledge that some prefer the terms “patients”, “consumers”, or “service users” to describe those who access health care, while others find some or all of these terms problematic. We have settled on the term “clients” since it seems the least problematic, while we recognize that some people might not like the term.

3 Throughout the article, we use the term “Indigenous” in relation to First Nations, Métis, and Inuit peoples in a desire to maintain consistent terminology. We recognize that the term “Indigenous” does not capture the diversity of distinct histories, languages, cultures, and experiences of the many First Nations, Métis, and Inuit peoples.
